# An Ingeniously Designed Skin Lesion Classification Model Across Clinical and Dermatoscopic Datasets

**DOI:** 10.3390/diagnostics15162011

**Published:** 2025-08-11

**Authors:** Ying Huang, Zhishuo Zhang, Xin Ran, Kaiwen Zhuang, Yuping Ran

**Affiliations:** 1 Department of Dermatovenereology, West China Hospital, Sichuan University, Chengdu 610041, China; hy863676590@sina.com (Y.H.); kaiwenzhuang@163.com (K.Z.); 2 School of Information and Software Engineering, University of Electronic Science and Technology of China (UESTC), Chengdu 610054, China

**Keywords:** skin lesion classification, hybird feature extraction, adaptively learnable activation function

## Abstract

**Background**: Skin cancer diagnosis faces critical challenges due to the visual similarity of lesions and dataset limitations. **Methods**: This study introduces HybridSkinFormer, a robust deep learning model designed to classify skin lesions from both clinical and dermatoscopic images. The model employs a two-stage architecture: a multi-layer ConvNet for local feature extraction and a residual-learnable multi-head attention module for global context fusion. A novel activation function (StarPRelu) and Enhanced Focal Loss (EFLoss) address neuron death and class imbalance, respectively. **Results**: Evaluated on a hybrid dataset (37,483 images across nine classes), HybridSkinFormer achieved state-of-the-art performance with an overall accuracy of 94.2%, a macro precision of 91.1%, and a macro recall of 91.0%, outperforming nine CNN and ViT baselines. **Conclusions**: Its ability to handle multi-modality data and mitigate imbalance highlights its clinical utility for early cancer detection in resource-constrained settings.

## 1. Introduction

The integumentary system as the body’s primary barrier against external insults plays a multifaceted role in maintaining homeostasis, encompassing protective, thermoregulatory, and metabolic functions [1]. As the largest organ, the skin—comprising the epidermis, dermis, and hypodermis—serves as a dynamic interface that safeguards against pathogens, mechanical stress, and ultraviolet (UV) radiation [2,3]. Despite its resilience, prolonged exposure to environmental stressors, genetic predispositions, and behavioral factors (e.g., unprotected sun exposure) elevate the risk of pathological transformations, including oncogenesis [4]. Skin cancer, characterized by uncontrolled proliferation of epidermal or melanocytic cells, ranks among the most prevalent malignancies worldwide, with melanoma alone accounting for the majority of skin cancer-related deaths [5].

Early detection of skin cancer is critical, as timely intervention can improve survival rates by up to 70%.

Advancements in deep learning (DL) have revolutionized medical image analysis, offering data-driven solutions to overcome the limitations of conventional diagnostics [6]. Convolutional neural networks (CNNs), the cornerstone of visual feature extraction, have demonstrated efficacy in skin lesion classification by automating the identification of discriminative patterns such as color heterogeneity and border irregularity. Works such as [7,8] leverage DenseNet and EfficientNet to learn deep features between lesion subtypes from datasets like HAM10000 or ISIC 2019. However, CNNs are inherently constrained by their local receptive field architecture, which limits their ability to model long-range spatial dependencies critical for capturing global lesion context [9].

Vision transformers (ViTs) [10,11], inspired by natural language processing [12], address this gap through self-attention mechanisms that enable global contextual modeling. By partitioning images into patch embeddings and encoding inter-patch relationships, ViTs such as DeiT [13] and Swin-Transformer [14] have shown promise in capturing holistic lesion characteristics, such as asymmetrical growth patterns and structural complexity. Yet, traditional ViTs suffer from high computational overhead, rendering them impractical for real-time applications or deployment on mobile IoT devices, such as portable dermatoscopes [15].

However, the current application of deep classification networks for skin disease categorization and benign–malignant lesion discrimination faces three key challenges:

- Single-Modality Dependency: Existing models are exclusively trained and tested on datasets containing only one type of image data (either dermatoscopic lesion images or clinical high-definition images). This restricts their applicability to a single imaging modality, limiting real-world usage scenarios and reducing diagnostic confidence. For instance, a model trained solely on dermatoscopic images may fail to generalize to clinical photographs with different lighting and magnification.
- Severe Class Imbalance: All datasets exhibit significant imbalance in the number of samples across lesion categories. During training, models tend to overfit to majority classes, forgetting features of minority classes. This imbalance introduces bias, causing models to favor predicting dominant categories and underperforming in rare lesion detection. For example, melanocytic nevi (with abundant samples) may overshadow vascular lesions (with scarce samples), leading to misdiagnosis of rare conditions.
- Lightweight Model Design for Resource-Constrained Systems: Developing a practical lightweight model that balances local feature extraction (e.g., texture details) and global context awareness (e.g., lesion architecture), while being deployable on medical IoT chips with limited computational resources, remains unsolved. Current models either excel in accuracy but are too heavy for edge devices or are lightweight but sacrifice multi-scale feature learning.

### Major Contributions

To address the aforementioned challenges, the key contributions of this work are succinctly summarized as follows:

- Hybrid Dataset Construction: We constructed a hybrid dataset comprising high-definition local clinical lesion images and dermatoscopic images using multi-center public datasets. This dataset is designed to train a lesion disease recognition and screening model capable of handling both clinical images and dermatoscopic photography, addressing the limitation of single-modality models and expanding applicability across diverse clinical scenarios.
- HybridSkinFormer Model Architecture: To address the challenge of classifying multi-type lesion images, we propose a deep recognition model called HybridSkinFormer. This model employs a two-stage feature extraction strategy: (a) Local feature extraction via multi-layer ConvNet, capturing fine-grained details such as texture and color variations in lesions. (b) Global feature fusion using a residual-learnable multi-head attention module, enabling the model to integrate contextual information across the entire image.
  Additionally, we introduce a novel activation function, StarPRelu, to mitigate the “dying ReLU” problem by preserving negative gradient flow. To tackle class imbalance in the training data, we enhance the Focal Loss with an adaptive scaling mechanism, resulting in the Enhanced Focal Loss (EFLoss). EFLoss dynamically adjusts loss weights based on class sample ratios and current loss values, improving minority class representation during training.
- Adequate Experimental Validation: The trained model was evaluated on a test set disjoint from the training data and compared against state-of-the-art lightweight deep image classification models. Results demonstrate that HybridSkinFormer achieves optimal or comparable performance across all metrics, highlighting its effectiveness in multi-modality lesion classification and robustness to class imbalance.

The proposed auxiliary skin lesion discrimination model can be deployed on portable dermatoscopic devices, enabling primary hospital physicians to access auxiliary support for differential diagnosis.

## 2. Related Works

The rapid advancements in deep learning (DL) have profoundly influenced the domain of dermatological imaging and skin lesion detection, with numerous studies exploring innovative methodologies to enhance diagnostic accuracy and support medical training through advanced DL models. This review synthesizes key findings from recent research, focusing on convolutional neural networks (CNNs) and transfer learning (TL) frameworks for improved lesion detection and classification.

Chang et al. [16] employed the self-developed InceptionResNetV2 model to enhance image resolution and analytical capabilities on the HAM10000 dataset. Similarly, Khattar et al. [17], Shete et al. [18], Garg et al. [19], Shehzad et al. [20], and Ahmad et al. [21] devised the customized InceptionResNetV2-based ConvNet model to analyze and classify the skin lesions, focusing on optimizing the training process for dermatological education and improving lesion discrimination accuracy.

Satapathy et al. [22] utilized a Capsule Network (CapsNet) for skin lesion classification on the HAM10000 dataset. Unlike traditional convolutional neural networks (CNNs), the CapsNet architecture is specifically designed to model spatial hierarchies and preserve positional information, enabling more precise representation of lesion structures. Through this innovative approach, the model demonstrated enhanced capability in capturing complex spatial relationships within dermatoscopic images, offering a promising alternative for addressing the challenges of lesion classification caused by visual feature variability.

Xie et al. [23] conducted an exploration into the application of diverse CNN architectures on the Xiangya–Derm dataset. Nevertheless, challenges including dataset imbalance and the clinical similarity among disease categories highlighted the necessity for advanced data management strategies and optimized model training methods to reduce classification errors.

Anjum et al. [24] presented a three-stage comprehensive framework that employs models such as YOLOv2, ResNet-18, and Ant Colony Optimization (ACO) to achieve high-precision skin lesion detection and classification. While this framework demonstrates robust performance, it necessitates substantial computational resources, which may limit its applicability in resource-constrained environments. Goyal et al. [25] systematically reviewed diverse deep learning methodologies, highlighting ensemble techniques like DeeplabV3+ and Mask R-CNN. These approaches integrate semantic and instance segmentation to address noisy annotations and enhance model robustness, though they are characterized by high computational complexity and require meticulous preprocessing steps. Natasha Nigar et al. [26] emphasized the critical role of interpretability by integrating ResNet-18 with the LIME framework to generate visual explanations for predictions, thereby fostering clinical trust in AI-driven diagnostics. However, their approach is constrained by reliance on a single dataset and occasional inconsistencies in LIME-generated explanations. Collectively, these studies illustrate the dual promise and challenges of deploying deep learning models in dermatological diagnostics, particularly in balancing accuracy, interpretability, and computational efficiency.

Bian et al. [27] investigated the effectiveness of Multi-View Filtered Transfer Learning (MFTL) on ISIC 2017 and HAM10000 datasets, demonstrating notable performance improvements through selective sample distillation using Wasserstein distance. However, the study highlighted computational complexity and preprocessing hurdles as potential obstacles to broad clinical deployment.

Hosny et al. [28] proposed a novel deep inherent learning method for multi-class skin lesion classification. Combining multiple convolution filters and inherent blocks, the model overcomes image shortages and degradation. Using the HAM10000 dataset, it achieved 92.89% accuracy. Explainable AI techniques like occlusion sensitivity and feature visualization validated its ability to accurately detect lesion areas, outperforming prior methods in specificity and precision while providing transparent decision-making processes.

Naeem et al. [29] provided a comprehensive review of CNN-based melanoma detection methods, underscoring the pivotal role of diverse, large-scale datasets in enhancing model robustness. The review identified challenges in dataset diversity, computational efficiency, and model interpretability as critical areas for future research to elevate the clinical utility of AI-driven dermatological diagnostics.

Thurnhofer-Hemsi et al. [30] proposed an ensemble CNN approach with test-time shifting to address dataset imbalance, achieving superior results on the challenging HAM10000 dataset. Despite these advancements, the study acknowledged the computational burdens of ensemble learning and the need to optimize model scalability for heterogeneous clinical environments.

Liu et al. [31] introduced a multi-scale combined efficient channel attention module-empowered skin disease classification network. It improves the pyramid segmentation attention module by replacing the Squeeze-and-Excitation (SE) module module with the Efficient Channel Attention (ECA) and uses an inverted residual structure to enhance multi-scale feature extraction. Tested on ISIC2019 and HAM10000 datasets, the model achieves 77.6% and 88.2% accuracy, respectively, outperforming baseline models like ResNet and EPSANet.

Recently, Burhanettin Ozdemir and Ishak Pacal [32] devised a novel model combining ConvNeXtV2 blocks and separable self-attention, achieving 93.48% accuracy on ISIC 2019, outperforming over 20 state-of-the-art models with its efficient, hybrid architecture.

In summary, recent investigations highlight substantial progress in skin lesion classification via the integration of diverse datasets, sophisticated model architectures, and innovative methodologies like transfer learning and attention mechanisms. Notwithstanding these breakthroughs, challenges pertaining to dataset heterogeneity, computational complexity, and model interpretability remain as pivotal areas for future research. Addressing these challenges is essential to enhance the clinical applicability and trustworthiness of AI-powered diagnostic tools in dermatology, ensuring their effective deployment in real-world healthcare settings.

## 3. Materials and Methods

In this section, we introduce the dataset employed for training and testing, as well as the deep fitting model designed for fine-grained skin disease classification.

### 3.1. Datasets

**Figure 1:**
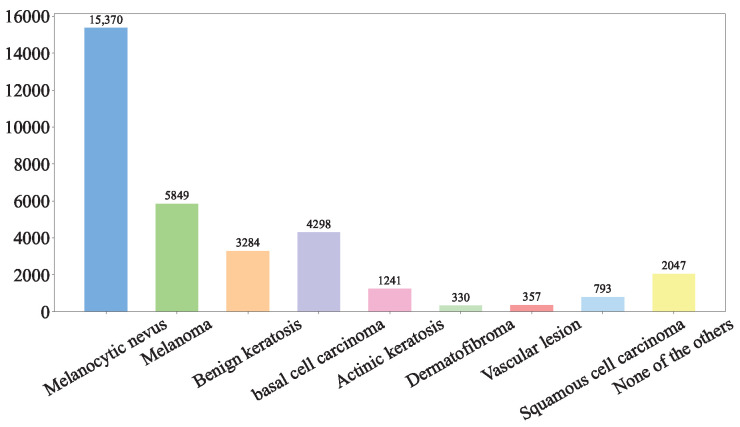
ISIC Challenge 2019 Dataset.

**Figure 2:**
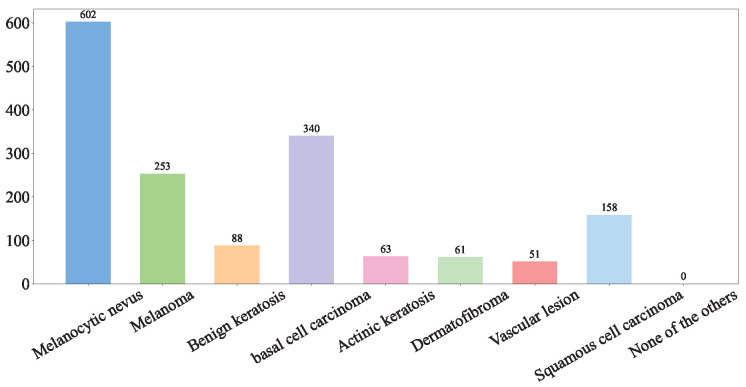
HIBA 2019–2022 dataset.

**Figure 3:**
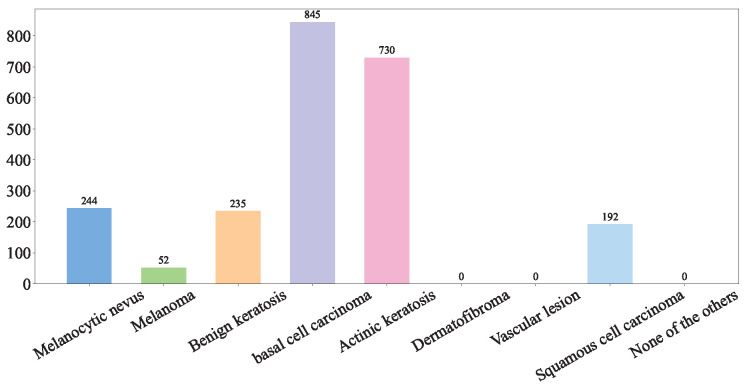
PAD-UFES-20 dataset.

This study leverages the following datasets for training and testing:

(1) ISIC Challenge 2019 Dataset: The ISIC 2019 dataset (https://challenge.isic-archive.com/data/#2019 (accessed on 8 August 2025)) is sourced from multiple international clinical centers and dermatological databases. The images are meticulously acquired using a diverse array of high-resolution dermatoscopes. In total, the dataset encompasses 33,569 skin lesion images. These images comprehensively depict a wide spectrum of skin conditions, including melanoma (MEL), basal cell carcinoma (BCC), and squamous cell carcinoma (SCC). The ISIC Challenge 2019 Dataset represents a comprehensive superset encompassing the HAM10000 [33], ISIC 2018 [8], and Bcn20000 datasets [34]. The class distribution of the skin lesions in ISIC Challenge 2019 dataset is shown in Figure 1.

(2) Hospital Italiano de Buenos Aires Skin Lesions Images 2019–2022 (HIBA 2019–2022): The Hospital Italiano de Buenos Aires Skin Lesions Images 2019–2022 (HIBA 2019–2022) dataset [35] is sourced from institutional records spanning 2019 to 2022. It contains information on patients with common skin lesions who visited the Dermatology Department during this period, along with their corresponding clinical and dermoscopy images. Three expert dermatologists carefully selected the cases included in the dataset. Clinical images were captured using the smartphones of the attending professionals. Dermoscopy images were obtained through a variety of professional dermatoscope devices, including the VL-7EX II video microscope (Scalar Corporation, Tokyo, Japan), Dermagraphix Mirror 7 (Canfield Scientific, Parsippany, NJ, USA), FotoFinder-compatible cameras such as the Medicam 1000s and Medicam 800Hd, and dermoscopic attachments for smartphones. The class distribution of the skin lesions in HIBA 2019–2022 dataset is shown in Figure 2.

(3) PAD-UFES-20: The PAD-UFES-20 dataset [36] consists of clinical images of skin lesions collected from various smartphone devices, paired with corresponding patient clinical information. Comprising 2298 samples, it covers six different types of skin lesions, including three forms of skin cancer and three common skin diseases. The distribution of the dataset can be visualized in Figure 3.

**Figure 4:**
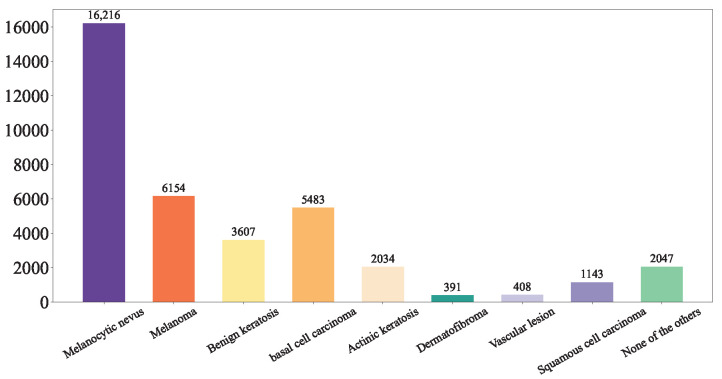
Our Integrated Dataset.

This study integrated the ISIC Challenge 2019 Dataset, the Hospital Italiano de Buenos Aires Skin Lesions Images (2019–2022), and the PAD-UFES-20 Dataset to form a comprehensive database of common benign and malignant skin diseases, incorporating 37,483 both clinical and dermoscopic images. This database encompasses nine distinct diagnostic categories: Melanoma (MEL), Melanocytic nevus (NV), Basal cell carcinoma (BCC), Actinic keratosis (AK), Benign keratosis (BKL, including solar lentigo/seborrheic keratosis/lichen planus-like keratosis), Dermatofibroma (DF), Vascular lesion (VASC), Squamous cell carcinoma (SCC), and Unknown (UNK). The detailed of our integrated dataset is shown in Figure 4.

**Figure 5:**
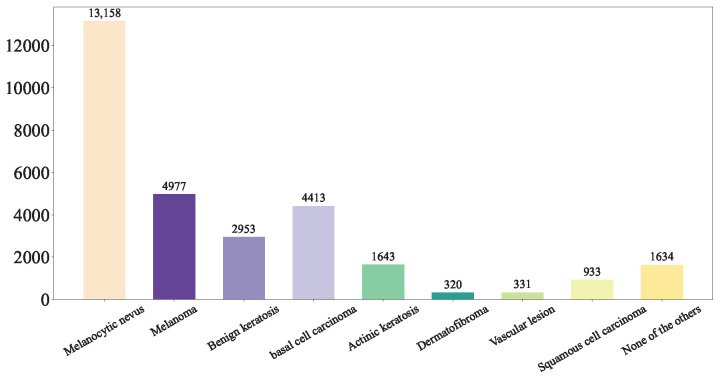
Our Training Set.

**Figure 6:**
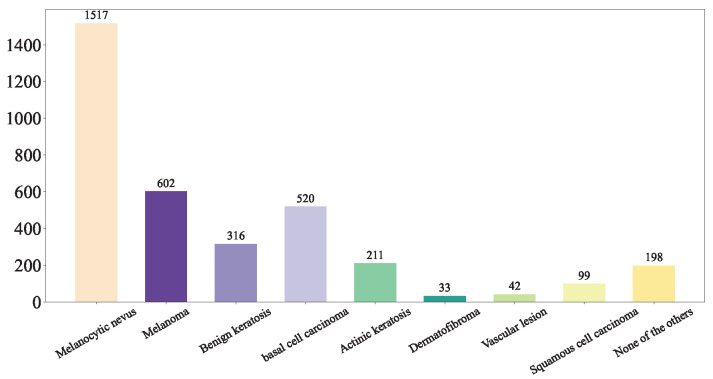
Our Test Set.

**Figure 7:**
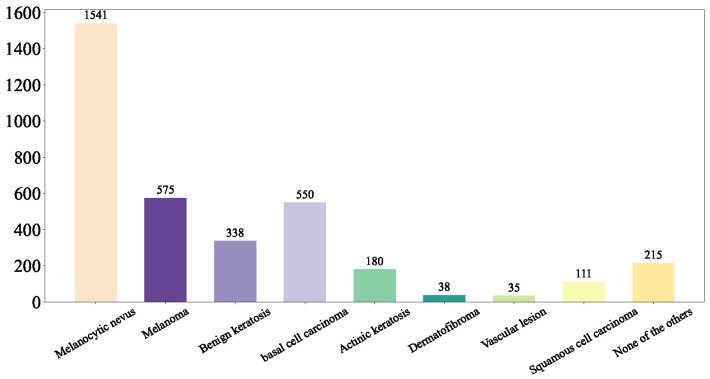
Our Validation Set.

First, we shuffled the data and extracted the training set Figure 5, test set Figure 6, and validation set Figure 7 using a stratified random sampling method for each category. This ensured the mutual exclusivity and randomness among the training set, test set, and validation set, while also guaranteeing that the number of samples in each category basically conforms to the distribution of the original overall dataset.

In total, 80% of the images was selected as the training set Figure 5, while the remaining 20% of the dataset was evenly divided into the validation set Figure 7 and the test set Figure 6.

### 3.2. Data Preprocessing and Augmentation

To improve the generalization and accuracy of our model, we perform the following preprocessing and augmentation on images.

**Figure 8:**
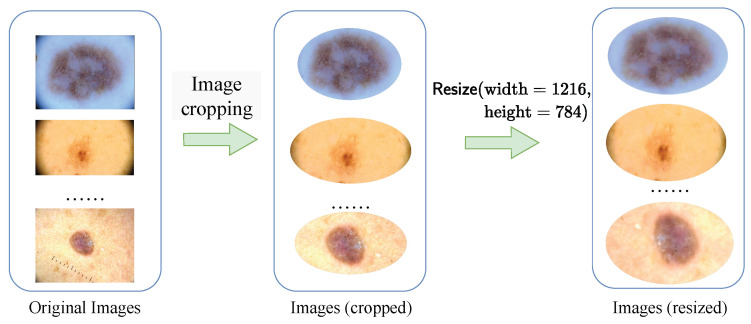
The preprocessing flow.

(1) Data Preprocessing: We meticulously crop each image in our integrated dataset (including training set, validation set and test set). We assign the skin background mucosa and noises irrelevant to lesions to (r,g,b,α)=(0,0,0,0). This step is essential to eliminate normal skin mucosa in the surrounding background and extraneous noise information, ensuring that the pathological skin lesions were centered within the images. To streamline the training and testing procedures, all images are standardized to a fixed resolution of (height=784, width=1216). This standardization is pivotal in enabling effective and consistent evaluation across different models and experiments. The preprocessing flow is depicted in Figure 8.

(2) Data Augmentation: To enhance the robustness of the trained discriminative model, during the training process, the data read from the training set undergoes operations such as (a) brightness adjustment, (b) contrast enhancement, and (c) image flipping. These operations are carried out to augment the diversity of the training data, which is crucial for improving the generalization ability and stability of the discriminative model in task of lesion recognition and classification.

(a) Brightness adjustment: Through gamma correction transformation (1), lesion images with different brightness levels can be generated to simulate the variations under different exposure parameters of the dermatoscope.

(1) $$\begin{array}{c} g(x,y)=\gamma f(x,y) \end{array}$$

where f(x,y) represents the pixel value of the input image at the position (x,y), and g(x,y) represents the pixel value of the output image at the position (x,y). γ is the adjustment coefficient. By setting different values of γ, the brightness of the image can be adjusted. When γ>1, the image becomes brighter; when 0<γ<1, the image becomes darker.

(b) Contrast enhancement: By using the hierarchical contrast stretching algorithm (2), we can simulate the skin lesion images captured under different lighting conditions in real-world scenarios.

(2) $$\begin{array}{c} g\left(x,y\right)=\left\{\begin{array}{cc} z_{1}f\left(x,y\right), & f\left(x,y\right)<r_{1}\\ z_{2}\left(f\left(x,y\right)-r_{1}\right)+s_{1}, & r_{1}\leq f\left(x,y\right)<r_{2}\\ z_{3}\left(f\left(x,y\right)-r_{2}\right)+s_{2}, & f\left(x,y\right)\geq r_{2} \end{array}\right. \end{array}$$

where f(x,y) denotes the pixel value of the input image at the position (x,y), and g(x,y) represents the pixel value of the output image at the position (x,y). $s_{1}$, $s_{2}$, $r_{1}$, and $r_{2}$ are user-defined parameters. Additionally, $z_{1}=s_{1}/r_{1}$, $z_{2}=\left(s_{2}-s_{1}\right)/\left(r_{2}-r_{1}\right)$ and $z_{3}=\left(L-s_{2}\right)/\left(L-r_{2}\right)$, *L* represents the maximum range of gray-level values.

- When the pixel value f(x,y) is less than $r_{1}$, the output pixel value g(x,y) is equal to $z_{1}$ multiplied by f(x,y), which enhances the contrast of darker regions.
- When $r_{1}\leq f\left(x,y\right)\leq r_{2}$, the output pixel value g(x,y) is calculated as $z_{2}\left(f\left(x,y\right)-r_{1}\right)+s_{1}$. This linear transformation adjusts the contrast of the middle-gray-level region.
- When $f\left(x,y\right)\geq r_{2}$, the output pixel value g(x,y) is $z_{3}\left(f\left(x,y\right)-r_{2}\right)+s_{2}$, which adjusts the contrast of brighter regions.

In this way, the details of the image can be made clearer, enhancing the visual effect of the image and facilitating subsequent image processing tasks.

(c) Image flipping: By randomly performing horizontal or vertical flips on the input skin lesion images, we can simulate the images captured by the dermatoscope lens at different rotation angles.

### 3.3. HybridSkinFormer Model

#### 3.3.1. Global Framework

**Figure 9:**
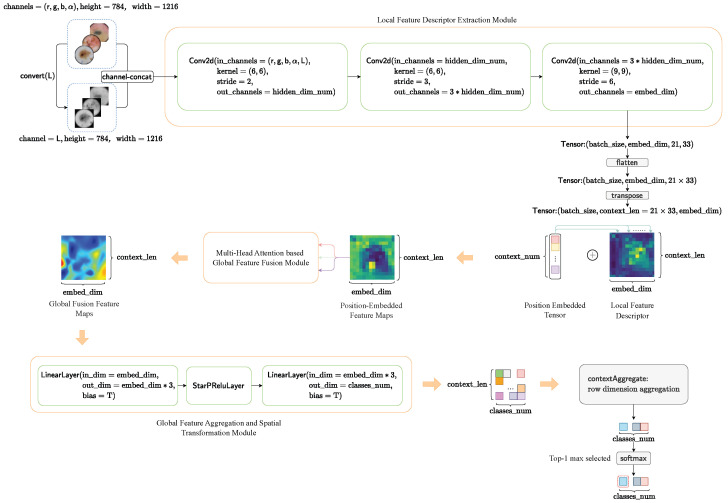
The framework of our proposed HybridSkinFormer.

The global framework of our proposed HybridSkinFormer is depicted in Figure 9.

First, we convert the pre-processed (r,g,b,α)-skin lesion image into a grayscale image using convert(″L″) and perform channel aggregation. As a result, an aggregated five-channel (r,g,b,α,L) original feature matrix is obtained.

(3) $$\begin{array}{cc} \; & \mathrm{skinimgL}=\mathrm{skinimg}.\mathrm{convert}(L) \end{array}$$

(4) $$\begin{array}{cc} \; & \mathrm{skinimgRGBAL}=\mathrm{channelconcat}(\mathrm{skinimg},\mathrm{skinimgL})\to \mathrm{Tensor}(5,784,1216) \end{array}$$

Subsequently, we extract local features from the five-channel (r,g,b,α,L) original feature matrix through three convolutional layers with gradually increasing kernel scales. This process yields a local high-dimensional feature matrix.

(5) $$\begin{array}{cc} X=\mathrm{Conv}2d & (\mathrm{input}=\mathrm{skinimgRGBAL},\mathrm{inchannels}=(r,g,b,\alpha ,L),\\ \; & \mathrm{kernel}=(6,6),\mathrm{stride}=2,\mathrm{outchannels}=\mathrm{hidden}\_\dim\_\mathrm{num}) \end{array}$$

(6) $$\begin{array}{cc} X=\mathrm{Conv}2d & (\mathrm{input}=X,\mathrm{inchannels}=\mathrm{hidden}\_\dim\_\mathrm{num},\\ \; & \mathrm{kernel}=(6,6),\mathrm{stride}=3,\mathrm{outchannels}=\mathrm{hidden}\_\dim\_\mathrm{num}*3) \end{array}$$

(7) $$\begin{array}{cc} \mathrm{LocalX}=\mathrm{Conv}2d & (\mathrm{input}=X,\mathrm{inchannels}=\mathrm{hidden}\_\dim\_\mathrm{num}*3,\\ \; & \mathrm{kernel}=(9,9),\mathrm{stride}=6,\mathrm{outchannels}=\mathrm{embed}\_\dim)\\ \to \mathrm{Tensor} & \left(\mathrm{batchsize},\mathrm{embed}\_\dim,21,33\right) \end{array}$$

Then, we flatten and transpose the local feature matrix to obtain the feature context feature matrix. We use a learnable tensor to perform positional encoding on the context feature matrix. Next, we input the position-embedded context feature matrix into the global feature fusion module with a sequence of multi-layer multi- head attention feature fusion layer for. The output is a globally fused feature tensor.

(8) $$\begin{array}{cc} \mathrm{LocalX} & =\mathrm{flatten}(\mathrm{LocalX})\to \mathrm{Tensor}(\mathrm{batchsize},\mathrm{embed}\_\dim,21\times 33)\\ \mathrm{GlobalX} & =\mathrm{transport}(\mathrm{GlobalX})\to \mathrm{Tensor}(\mathrm{batchsize},\mathrm{context}\_\mathrm{num}=21\times 33,\mathrm{embed}\_\dim)\\ \mathrm{GlobalX} & ={\mathrm{MultiheadAttenLayer}}_{i}\left(\mathrm{GlobalX},\mathrm{headnum}=\mathrm{embed}\_\dim//3\right),\\ \to \mathrm{Tensor} & (\mathrm{batchsize},\mathrm{context}\_\mathrm{num}=21\times 33,\mathrm{embed}\_\dim),\mathrm{where}\;\;i=1,\ldots ,\mathrm{attenlayernum} \end{array}$$

After that, we input the output global feature fusion tensor into a multi-layer perceptron layer to project the features at each position into the classification weight space.

(9) $$\begin{array}{cc} \mathrm{ClassProjX}= & \mathrm{LinearLayer}(\mathrm{GlobalX},\\ \; & \mathrm{indim}=\mathrm{embed}\_\dim,\mathrm{outdim}=\mathrm{embed}\_\dim*3,\mathrm{bias}=T) \end{array}$$

(10) ClassProjX=StarPReluLayer(ClassProjX)

(11) $$\begin{array}{cc} \mathrm{ClassProjX}= & \mathrm{LinearLayer}(\mathrm{ClassProjX},\\ \; & \mathrm{indim}=\mathrm{embed}\_\dim*3,\mathrm{outdim}=\mathrm{classes}\_\mathrm{num},\mathrm{bias}=T)\\ \to & \mathrm{Tensor}(\mathrm{batchsize},\mathrm{context}\_\mathrm{num}=21\times 33,\mathrm{classes}\_\mathrm{num}) \end{array}$$

Finally, we sum the classification weights at each position to obtain an overall classification weight vector and then pass the overall classification weight vector through a softmax layer to obtain the top 1 probability of the skin lesion disease classification category.

(12) $$\begin{array}{c} \vec{\mathrm{clsprob}}=\mathrm{softmax}\left(\mathrm{sum}\left(\mathrm{ClassProjX},\dim=\mathrm{row}\right)\right)\to \mathrm{Vector}\left(\mathrm{classes}\_\mathrm{num}\right) \end{array}$$

The forward flow of our HybridSkinFormer is depicted in Algorithm 1.
**Algorithm 1:** HybridSkinFormer Forward Framework
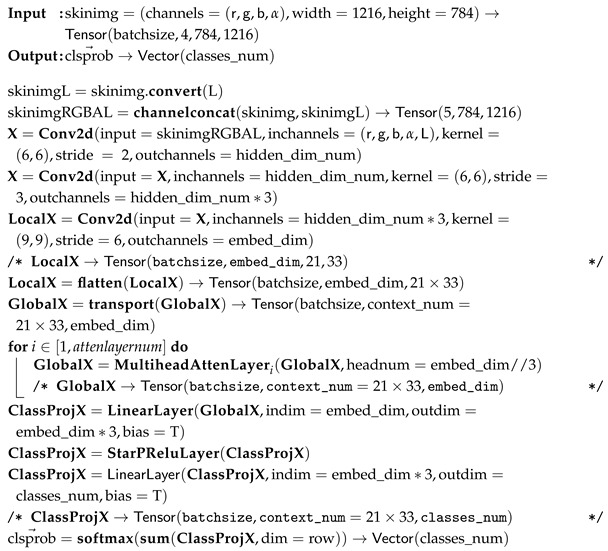


#### 3.3.2. Global Feature Fusion Module

**Figure 10:**
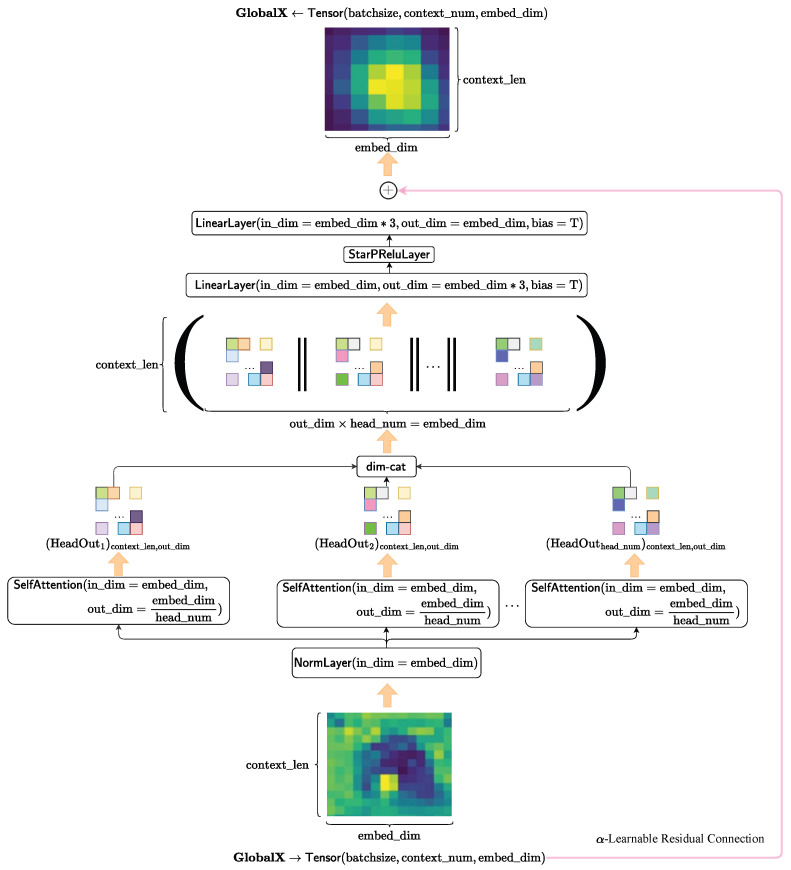
The framework of the multi-head attention layer.

The global feature fusion module in our HybridSkinFormer is composed of a deep sequence of the multi-head attention module with α-learnable residual connection and pre-normalization mechanism presented in Figure 10.

First, we perform pre-normalization on the input tensor along the feature dimension.

(13) $$\begin{array}{cc} X & =\mathrm{NormLayer}(\mathrm{input}=\mathrm{GlobalX},\mathrm{in}\_\dim=\mathrm{embed}\_\dim)\\ \; & \to \mathrm{Tensor}(\mathrm{batchsize},\mathrm{context}\_\mathrm{num},\mathrm{embed}\_\dim) \end{array}$$

Subsequently, we feed the normalized tensor into multiple attention heads in parallel. Then, we concatenate the outputs of each attention head along the feature dimension to obtain the feature aggregation of multiple attention heads.

(14) $$\begin{array}{cc} {\mathrm{HeadOut}}_{k}= & {\mathrm{SelfAttentionModule}}_{k}(X,\\ \; & \mathrm{in}\_\dim=\mathrm{embed}\_\dim,\mathrm{out}\_\dim=\mathrm{embed}\_\dim//\mathrm{head}\_\mathrm{num}),\\ \to & \mathrm{Tensor}(\mathrm{batchsize},\mathrm{context}\_\mathrm{num},\mathrm{embed}\_\dim//\mathrm{head}\_\mathrm{num}),\\ & \mathrm{where}\;\;k=1,\ldots ,\mathrm{head}\_\mathrm{num} \end{array}$$

(15) $$\begin{array}{cc} X & =\mathrm{concat}(\left[{\mathrm{HeadOut}}_{1},\ldots ,{\mathrm{HeadOut}}_{k},\ldots ,{\mathrm{HeadOut}}_{\mathrm{head}\_\mathrm{num}}\right],\dim=col) \end{array}$$

After that, we input the attention-aggregated feature tensor into the spatial transformation layer to map the features to a new space.

(16) $$\begin{array}{cc} X & =\mathrm{LinearLayer}(X,\mathrm{in}\_\dim=\mathrm{embed}\_\dim,\mathrm{out}\_\dim=\mathrm{embed}\_\dim*3,\mathrm{bias}=T) \end{array}$$

(17) $$\begin{array}{cc} X & =\mathrm{StarPReluLayer}(X)\;\;\;\;\;\;\;\;\;\;\;\;\;\;\;\;\;\;\;\;\; \end{array}$$

(18) $$\begin{array}{cc} X & =\mathrm{LinearLayer}(X,\mathrm{in}\_\dim=\mathrm{embed}\_\dim*3,\mathrm{out}\_\dim=\mathrm{embed}\_\dim,\mathrm{bias}=T)\\ \; & \to \mathrm{Tensor}(\mathrm{batchsize},\mathrm{context}\_\mathrm{num},\mathrm{embed}\_\dim) \end{array}$$

Finally, we conduct an alpha-independent learnable residual connection between the original input tensor and the transformed feature tensor to obtain the feature fusion output of this layer.

(19) $$\begin{array}{cc} \mathrm{GlobalX} & =\alpha *\mathrm{GlobalX}+(1-\alpha )*X\to \mathrm{Tensor}(\mathrm{batchsize},\mathrm{context}\_\mathrm{num},\mathrm{embed}\_\dim) \end{array}$$

where α is a independent learnable parameter.

The forward flow of the multi-head attention module with α-learnable residual connection and pre-normalization mechanism in our HybridSkinFormer is depicted in Algorithm 2.
**Algorithm 2:** Multi-head attention module with α-learnable residual connection and pre-normalization
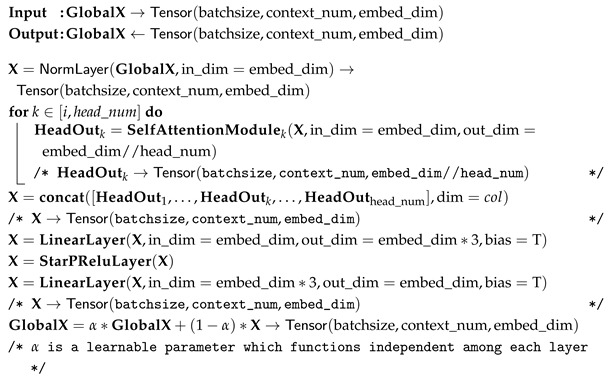


**Figure 11:**
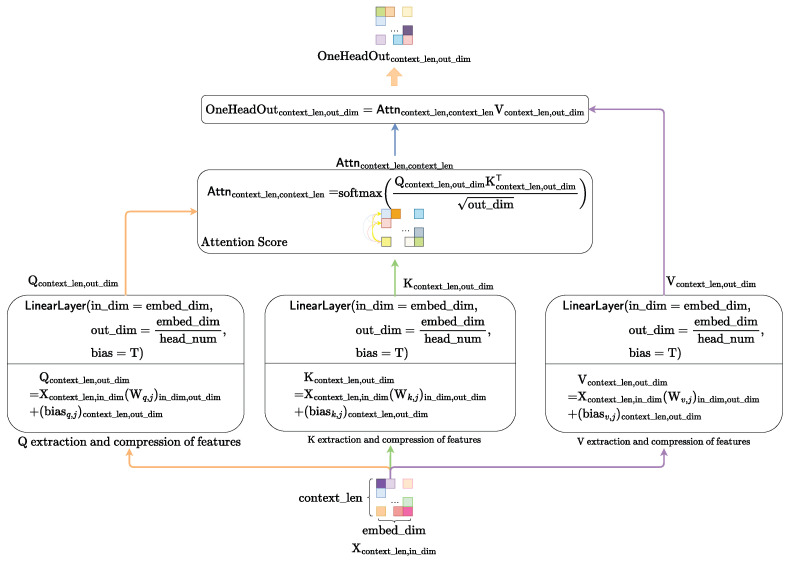
The computational flow of self-attention mechanism.

We adopt the QKV-softmax attention mechanism transferring from the Transformer to instantiate the SelfAttentionModule. The computational flow of the QKV-softmax attention mechanism is presented in Figure 11.

First, we compute the query matrix $Q_{\mathrm{context}\_\mathrm{len},\mathrm{out}\_\dim}$, key matrix $K_{\mathrm{context}\_\mathrm{len},\mathrm{out}\_\dim}$ and value matrix $V_{\mathrm{context}\_\mathrm{len},\mathrm{out}\_\dim}$ from context matrix $X_{\mathrm{context}\_\mathrm{len},\mathrm{in}\_\dim}$ through the linear space transformation.

(20) $$\begin{array}{cc} \; & Q_{\mathrm{context}\_\mathrm{len},\mathrm{out}\_\dim}=X_{\mathrm{context}\_\mathrm{len},\mathrm{in}\_\dim}{\left(W_{q,j}\right)}_{\mathrm{in}\_\dim,\mathrm{out}\_\dim}+{\left({\mathrm{bias}}_{q,j}\right)}_{\mathrm{context}\_\mathrm{len},\mathrm{out}\_\dim} \end{array}$$

(21) $$\begin{array}{cc} \; & K_{\mathrm{context}\_\mathrm{len},\mathrm{out}\_\dim}=X_{\mathrm{context}\_\mathrm{len},\mathrm{in}\_\dim}{\left(W_{k,j}\right)}_{\mathrm{in}\_\dim,\mathrm{out}\_\dim}+{\left({\mathrm{bias}}_{k,j}\right)}_{\mathrm{context}\_\mathrm{len},\mathrm{out}\_\dim} \end{array}$$

(22) $$\begin{array}{cc} \; & V_{\mathrm{context}\_\mathrm{len},\mathrm{out}\_\dim}=X_{\mathrm{context}\_\mathrm{len},\mathrm{in}\_\dim}{\left(W_{v,j}\right)}_{\mathrm{in}\_\dim,\mathrm{out}\_\dim}+{\left({\mathrm{bias}}_{v,j}\right)}_{\mathrm{context}\_\mathrm{len},\mathrm{out}\_\dim} \end{array}$$

Subsequently, we calculate the attention score using query matrix $Q_{\mathrm{context}\_\mathrm{len},\mathrm{out}\_\dim}$ and key matrix $K_{\mathrm{context}\_\mathrm{len},\mathrm{out}\_\dim}$.

(23) $$\begin{array}{c} {\mathrm{Attn}}_{\mathrm{context}\_\mathrm{len},\mathrm{context}\_\mathrm{len}}=\mathrm{softmax}\left(\frac{Q_{\mathrm{context}\_\mathrm{len},\mathrm{out}\_\dim}K_{\mathrm{context}\_\mathrm{len},\mathrm{out}\_\dim}^{T}}{\sqrt{\mathrm{out}\_\dim}}\right) \end{array}$$

Finally, using the calculated attention score ${\mathrm{Attn}}_{\mathrm{context}\_\mathrm{len},\mathrm{context}\_\mathrm{len}}$, we perform the weighted feature fusion for the value matrix $V_{\mathrm{context}\_\mathrm{len},\mathrm{out}\_\dim}$ to obtain the one-attention-head feature-fusion matrix ${\mathrm{HeadOut}}_{\mathrm{context}\_\mathrm{len},\mathrm{out}\_\dim}$.

(24) $$\begin{array}{c} {\mathrm{HeadOut}}_{\mathrm{context}\_\mathrm{len},\mathrm{out}\_\dim}={\mathrm{Attn}}_{\mathrm{context}\_\mathrm{len},\mathrm{context}\_\mathrm{len}}V_{\_\mathrm{len},\mathrm{out}\_\dim} \end{array}$$

### 3.4. Adaptive Activation Function

We define an adaptively learnable activation function StarPRelu, as shown in Equation (25), to simulate nonlinear transformations with different scales. The StarPRelu activation function can effectively prevent the issue of neuron death when the input signal is less than zero.

(25) $$\begin{array}{c} \mathrm{StarPRelu}(x)=\mathrm{PReLU}(x)\cdot (\psi |x|+\beta ) \end{array}$$

where PRelu is defined as (26).

(26) $$\begin{array}{c} \mathrm{PRelu}\left(x\right)=\left\{\begin{array}{cc} x & \mathrm{if}\;x\geq 0\\ \alpha x & \mathrm{if}\;x<0 \end{array}\right. \end{array}$$

**Figure 12:**
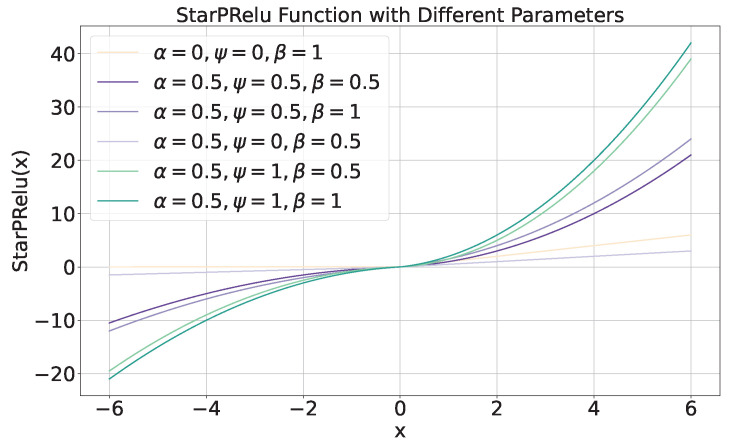
The introduced StarPRelu activation function with different parameters.

The introduced StarPRelu activation function with different parameters is presented in Figure 12. From Figure 12, we know that when parameters α=0, ψ=0, β=1, StarPRelu equals to ReLu.

### 3.5. Enhanced Focal Loss

To address the class imbalance issue in the training dataset of skin lesion images, we adopt the Category-Balanced Enhanced Focal Loss function EFLoss with reward and penalty mechanisms (27). This approach assigns smaller reward coefficients to categories with larger sample sizes and larger coefficients to those with smaller sizes, effectively mitigating the model’s classification bias towards majority classes during training.

(27) $$\begin{array}{c} \mathrm{EFLoss}\left({\vec{y}}_{i},{\vec{\hat{y}}}_{i}\right)=\sum_{j=1}^{\mathrm{classes}\_\mathrm{num}}-\beta \sigma \left(\left(\alpha_{j}\right)\right){\left(1-{\hat{y}}_{ij}\right)}^{\gamma }y_{ij}log\left({\hat{y}}_{ij}\right) \end{array}$$

where

- ${\vec{y}}_{i}=\left(y_{i1},\ldots ,y_{ij},\ldots ,y_{i,\mathrm{classes}\_\mathrm{num}}\right)$ is the ground truth probabilities vector of the sample *i*, $0\leq y_{ij}\leq 1$ is the groud truth probability of class *j*.
- ${\vec{\hat{y}}}_{i}=\left({\hat{y}}_{i1},\ldots ,{\hat{y}}_{ij},\ldots ,{\hat{y}}_{i,\mathrm{classes}\_\mathrm{num}}\right)$ is the predicted probabilities vector of the sample *i*, $0\leq {\hat{y}}_{ij}\leq 1$ is the predicted probability of class *j*.
- β, γ are the hyper-parameters. β>1 and 0≤γ<1.
- $\alpha_{j}$ is the scalar factor for each class. The larger the proportion of samples in a class *j*, the smaller the $\alpha_{j}$. We calculate the $\alpha_{j}=1-\frac{{\mathrm{class}\_\mathrm{num}}_{j}}{\mathrm{total}\_\mathrm{num}}$

When it comes to a deterministic classification task, the EFLoss can be simplified as (28).

(28) $$\begin{array}{c} \mathrm{EFLoss}\left({\vec{y}}_{i},{\vec{\hat{y}}}_{i}\right)=-\beta \sigma \left(\alpha_{\mathrm{truth}}\right){\left(1-{\hat{y}}_{i,\mathrm{truth}}\right)}^{\gamma }log\left({\hat{y}}_{i,\mathrm{truth}}\right) \end{array}$$

**Figure 13:**
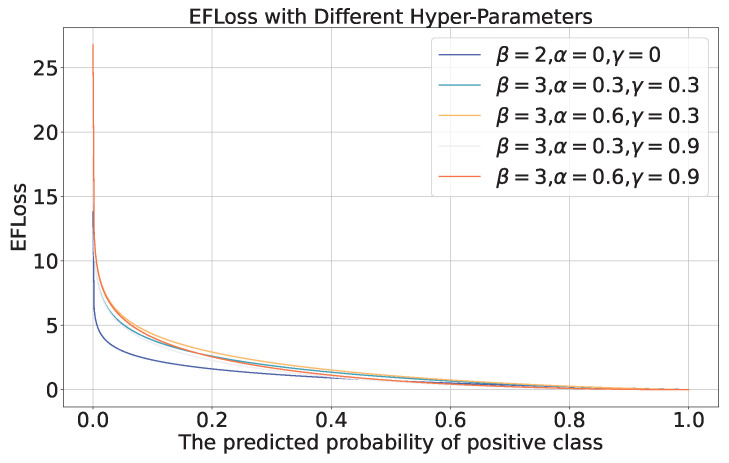
EFLoss with different hyper-parameters.

The EFLoss with different hyper-parameters is shown in Figure 13. When β=2, α=0 and γ=0, the proposed EFLoss degenerates into traditional Cross Entropy Loss (CELoss).

## 4. Experiment Evaluation and Discussion

### 4.1. Experimental Setup and Main Results

We employ the cloud computing resources of AutoDL (https://www.autodl.com/) to train our model. The settings of the software and hardware parameters as well as the hyperparameters are shown in Table 1. The best performance of our model is achieved at batchsize=10.

We utilize the function profile in util-library thop to calculate the parameter size and FLOPs of our HybridSkinFormer model. The parameter size of our HybridSkinFormer model is 310.599 K and the FLOPs is 817.265 M.

**Figure 14:**
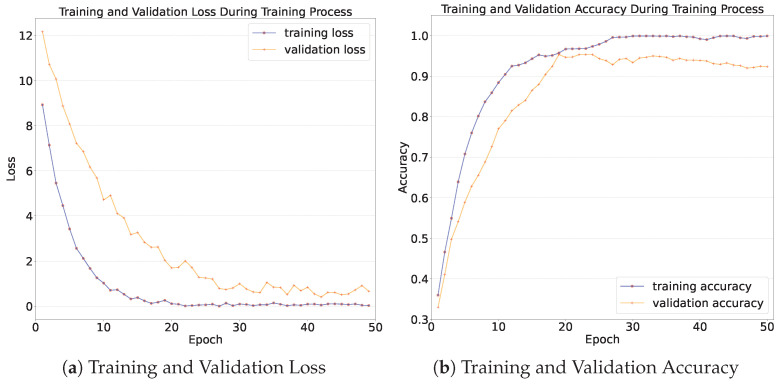
Loss and accuracy during training process.

The training and validation loss is presented in Figure 14a. As can be seen in Figure 14a, as the number of training epochs increases, the average training and validation losses per epoch exhibit a downward trend. The training loss stabilizes around the 20th epoch, reaching a value of 0.07 at the end of the training. The validation loss stabilizes around the 30th epoch, with a value of 0.18 at the end of the training.

The training and validation accuracy is shown in Figure 14b. As can be seen from Figure 14b, as the number of training epochs increases, both the training accuracy and the validation accuracy exhibit an upward trend. The upward trend is most prominent during the first 10 epochs. The training accuracy increases from 36% to 90%, and the validation accuracy increases from 36% to over 80%. After 30 epochs of training, both the training accuracy and the validation accuracy of the model tend to stabilize, which indicates that the model converges after 30 epochs. Eventually, after 50 epochs of training, the training accuracy reaches 99%, and the validation accuracy reaches 93%. This implies that the model has learned the high-dimensional features of different skin lesion categories and demonstrates good generalization performance on data that are outside the training dataset.

We apply the trained model to the test set to determine whether the model simply overfits and “records” the data it saw or truly learns the hidden features in each skin lesion image. In this way, we test the availability and generalization ability of the model in the real world.

**Figure 15:**
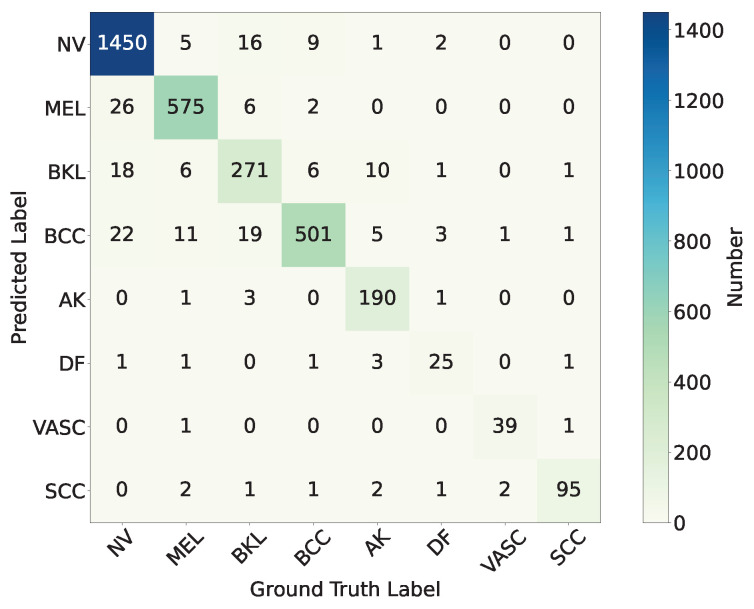
The confusion matrix of trained HybridSkinFormer on test set.

The final classification performance of the trained model on the test set is shown in the confusion matrix as Figure 15. According to the results of the confusion matrix shown in Figure 15, we calculate the precision, recall, F1-score, and Matthews Correlation Coefficient (MCC) for each category.

(29) $$\begin{array}{cc} \; & {\mathrm{Precision}}_{j}=\frac{{\mathrm{TP}}_{j}}{{\mathrm{TP}}_{j}+{\mathrm{FP}}_{j}},{\mathrm{Recall}}_{j}=\frac{{\mathrm{TP}}_{j}}{{\mathrm{TP}}_{j}+{\mathrm{FN}}_{j}},\forall j\in \left[1,\mathrm{classes}\_\mathrm{num}\right] \end{array}$$

(30) $$\begin{array}{cc} \; & F1_{j}=2\times \frac{{\mathrm{Precision}}_{j}\times {\mathrm{Recall}}_{j}}{{\mathrm{Precision}}_{j}+{\mathrm{Recall}}_{j}},\forall j\in \left[1,\mathrm{classes}\_\mathrm{num}\right] \end{array}$$

(31) $$\begin{array}{cc} \; & {\mathrm{MCC}}_{j}=\frac{{\mathrm{TP}}_{j}\times {\mathrm{TN}}_{j}-{\mathrm{FP}}_{j}\times {\mathrm{FN}}_{j}}{\sqrt{\left({\mathrm{TP}}_{j}+{\mathrm{FP}}_{j}\right)\left({\mathrm{TP}}_{j}+{\mathrm{FN}}_{j}\right)\left({\mathrm{TN}}_{j}+{\mathrm{FP}}_{j}\right)\left({\mathrm{TN}}_{j}+{\mathrm{FN}}_{j}\right)}},\forall j\in \left[1,\mathrm{classes}\_\mathrm{num}\right] \end{array}$$

In addition, we calculate the overall accuracy, (macro-mean) precision, recall, F1-score, and MCC value. The calculation formulas are as follows:

(32) $$\begin{array}{cc} \; & \mathrm{Acc}=\frac{\sum_{j=0}^{\mathrm{classes}\_\mathrm{num}}{\mathrm{TP}}_{j}}{\mathrm{total}\_\mathrm{num}} \end{array}$$

(33) $$\begin{array}{cc} \; & {\mathrm{Precision}}_{\mathrm{avg}}=\frac{\sum_{j=0}^{\mathrm{classes}\_\mathrm{num}}{\mathrm{Precision}}_{j}}{\mathrm{classes}\_\mathrm{num}} \end{array}$$

(34) $$\begin{array}{cc} \; & {\mathrm{Recall}}_{\mathrm{avg}}=\frac{\sum_{j=0}^{\mathrm{classes}\_\mathrm{num}}{\mathrm{Recall}}_{j}}{\mathrm{classes}\_\mathrm{num}} \end{array}$$

(35) $$\begin{array}{cc} \; & F1_{\mathrm{avg}}=\frac{\sum_{j=0}^{\mathrm{classes}\_\mathrm{num}}F1_{j}}{\mathrm{classes}\_\mathrm{num}} \end{array}$$

(36) $$\begin{array}{cc} \; & {\mathrm{MCC}}_{\mathrm{avg}}=\frac{\sum_{j=0}^{\mathrm{classes}\_\mathrm{num}}{\mathrm{MCC}}_{j}}{\mathrm{classes}\_\mathrm{num}} \end{array}$$

where ${\mathrm{TP}}_{j}$ is the true positive number of class *j*, ${\mathrm{FP}}_{j}$ is the false positive number of class *j*, ${\mathrm{FN}}_{j}$ is the false negative number of class *j*, ${\mathrm{TN}}_{j}$ is the true negative number of class *j*.

Precision reflects the proportion of true positive cases among the positive cases predicted by the model, and it focuses on measuring the accuracy of the model’s predictions. Recall emphasizes the degree to which the model covers positive samples. The recall rate for malignant classified lesions is of great significance, representing the model’s ability to screen and identify malignant skin lesions.

**Figure 16:**
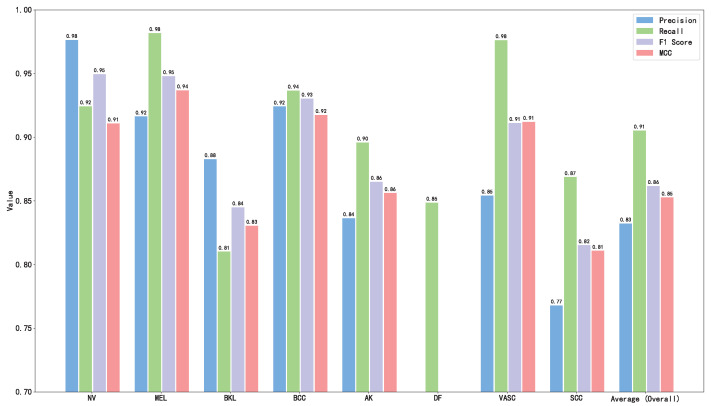
Performance metrics of our HybridSkinFormer on test set.

The calculation results are shown in Table 2 and Figure 16. As can be seen from Table 2 and Figure 16, the proposed model HybridSkinFormer achieved an overall accuracy of 94.2% on the test set, along with an overall (macro mean) precision of 91.2%, an overall (macro mean) recall of 91.0%, an overall (macro mean) F1-score of 0.911, and an overall (macro mean) MCC of 0.901.

According to the confusion matrix and Table 2: (a) Among the test samples of Melanocytic nevus (NV), 26 are misclassified as Melanoma (MEL), 18 are misclassified as Benign keratosis (BKL, including solar lentigo/seborrheic keratosis/lichen planus-like keratosis), and 22 are misclassified as Basal cell carcinoma (BCC). The precision is 97.8% and the recall is 95.6%. (b) Among the test samples of Melanoma (MEL), 11 are misclassified as Basal cell carcinoma (BCC), 6 are misclassified as Benign keratosis (BKL), and another 11 are misclassified as Basal cell carcinoma (BCC). The precision is 94.4% and the recall is 95.5%. (c) Among the test samples of Benign keratosis (BKL), 16 are misclassified as Melanocytic nevus (NV), 6 are misclassified as Melanoma (MEL), and 19 are misclassified as Basal cell carcinoma (BCC). The precision is 86.6% and the recall is 85.8%. (d) Among the test samples of Basal cell carcinoma (BCC), nine are misclassified as Melanocytic nevus (NV), six are misclassified as Benign keratosis (BKL). The precision is 89.0% and the recall is 96.3%. (e) Among Actinic keratosis (AK), 10 are misclassified as Benign keratosis (BKL), 5 are misclassified as Basal cell carcinoma (BCC). The precision is 97.4% and the recall is 90.0%. (f) Among the test samples of Dermatofibroma (DF), two are misclassified as Melanocytic nevus (NV), three are misclassified as Basal cell carcinoma (BCC). The precision is 78.1% and the recall is 75.8%. (g) Among Vascular lesion (VASC), only one is misclassified as Basal cell carcinoma (BCC). The precision is 95.1% and the recall is 92.9%. (h) Among the test samples of Squamous cell carcinoma (SCC), only four are misclassified. The precision is 91.3% and the recall is 96.0%.

In the test evaluation of this study, the model demonstrates excellent detection performance for all categories of malignant skin lesions, with the recall rate stably remaining above 95%. Specifically, the recall rate of Melanoma (MEL) reaches 95.5%, indicating that the model can successfully identify 95.5% of actual melanoma cases, effectively reducing the risk of missed diagnosis. The recall rate of Basal cell carcinoma (BCC) is 96.3%, suggesting that when detecting this common malignant skin tumor, the model can accurately capture the vast majority of cases. The recall rate of Squamous cell carcinoma (SCC) is 96.0%, also reflecting the high sensitivity of the model to this malignant lesion.

These results fully demonstrate that the model proposed in this study has excellent screening capabilities for malignant skin lesions. In clinical practice, a high recall rate can maximize the timely detection of malignant lesions, winning precious time for early intervention for patients and reducing the risk of delayed disease due to missed diagnosis. In addition, the stable performance of the model in different types of malignant lesions also provides solid data support and technical assurance for its clinical transformation and application in the field of early skin cancer diagnosis, and it is expected to become an effective auxiliary tool for dermatologists to quickly and accurately identify malignant esions.

Then, we analyze the reasons for the model’s misjudgments from the perspectives of clinical and skin lesion characteristics.

- *Confusion between Melanocytic Nevus (NV) and Melanoma (MEL):* In clinical or dermatoscopic images, some melanocytic nevi (NV) may exhibit characteristics indistinguishable from melanoma (MEL). In terms of color, early-stage or certain special types of MEL may show pigment distribution patterns similar to those of NV. Morphologically, atypical NV with local inflammatory cell infiltration may also present irregular shapes, consistent with the morphology of early-stage low-grade MEL.
- *Confusion between Dermatofibroma (DF) and Melanocytic Nevus (NV):* For dermatofibroma (DF) and melanocytic nevus (NV), pigmented DFs with tan or dark brown hues are nearly identical in color to NV, making accurate differentiation by color alone challenging. Morphologically, DF typically appears as a firm, elevated, oblate, or button-shaped nodule with a smooth surface; NV can also present as an elevated nodule with a smooth or slightly rough surface, resulting in morphological similarity.
- *Confusion between Dermatofibroma (DF) and Basal Cell Carcinoma (BCC):* Early-stage basal cell carcinoma (BCC) manifests as a slightly elevated, light yellow or pinkish small nodule with a firm texture on the local skin; DF may occasionally exhibit similar light yellow or pink coloration and firmness. Especially for smaller DFs, differentiation from early-stage BCC based solely on color and texture is difficult.
- *Confusion between Benign Keratosis (BKL) and Melanocytic Nevus (NV):* In terms of color, benign keratosis (BKL) encompasses a wide spectrum—skin-colored, light brown, dark brown, or black—overlapping considerably with NV’s color range. For example, seborrheic keratosis, a common type of BKL, often appears as brown or black flat papules or plaques, resembling pigmented NV and making color-based discrimination challenging. Morphologically, BKL typically presents as flat or slightly elevated lesions with clear borders and verrucous or papillomatous surfaces; NV can also be flat or elevated with well-defined margins. Congenital or atypical NV further blur morphological distinctions from BKL. Under dermatoscopy, both may exhibit non-specific features such as mottled or reticular pigmentation patterns, leading to diagnostic confusion in images.
- *Confusion between Benign Keratosis (BKL) and Basal Cell Carcinoma (BCC):* Basal cell carcinoma (BCC) often presents as a pearly or translucent papule/nodule with dilated surface capillaries. Some BKLs, such as actinic keratosis, may develop similar features during progression: mild elevation, rough texture, and vascular dilation. When occurring on the head and face—common sites for BCC—actinic keratosis closely mimics early BCC in appearance, increasing the risk of misdiagnosis. While dermatoscopic features like blue-gray globules or ulcers are typical of BCC, certain BKLs (e.g., seborrheic keratosis with comedo-like openings or milia cysts) may exhibit overlapping non-specific structures. Vascular dilation in both entities further complicates dermatoscopic differentiation.

### 4.2. Model Interpretability Evaluation

**Figure 17:**
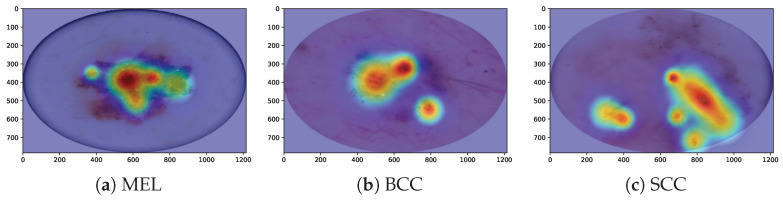
Model interpretability evaluation with Grad-CAM visualization technique.

We utilize Grad-CAM, a widely recognized visualization tool in research, to elucidate the critical features learned by our HybridSkinFormer model, thereby offering valuable support for subsequent classification decisions. In the visualization process, Grad-CAM first employs gradient calculation methods to generate feature maps for each category. Second, these feature maps are projected onto the original image, facilitating the identification of decision-making regions within the image. Owing to its linear nature, Grad-CAM can be implemented quickly and seamlessly across various neural networks. Finally, unlike methods that utilize “bounding boxes” to indicate the position of specific objects in classification images, Grad-CAM generates a pixel map associated with the target object. This approach yields more accurate, detailed, and high-fidelity visualization results. Figure 17 shows our HybridSkinFormer Model with Grad-CAM visualization technique for the sampled malignant lesion, e.g., MEL, SCC, and BCC. Through the Grad-CAM visualization technique, it can be intuitively observed that our discriminative model is able to accurately focus its attention on the regions with prominent features of malignant lesions.

### 4.3. Ablation Study

**Figure 18:**
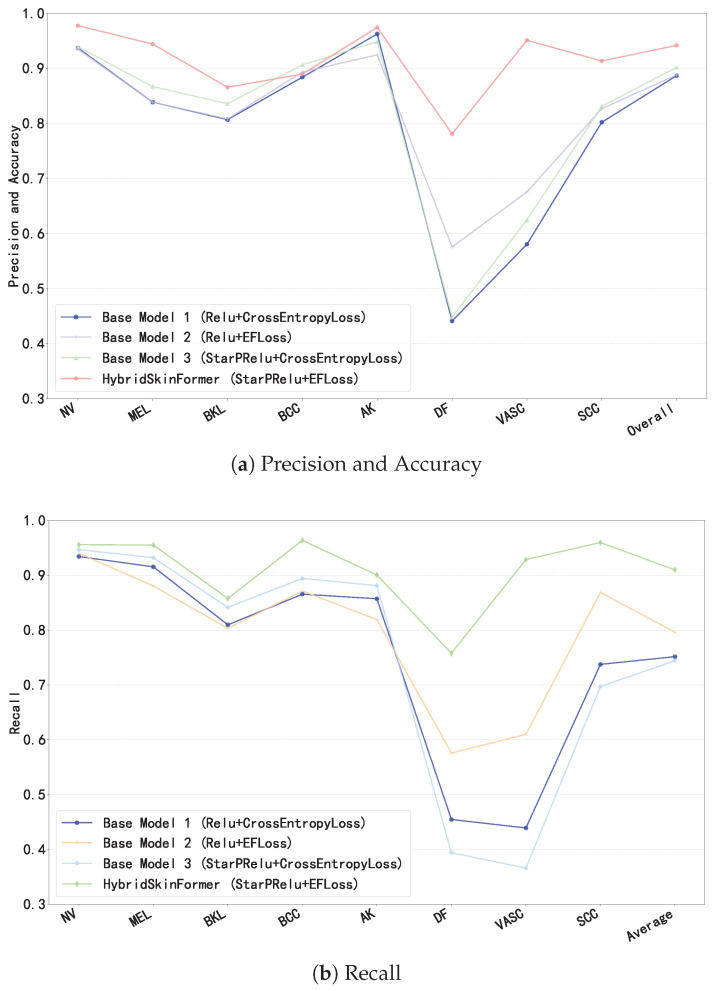
Ablation study.

To evaluate the effectiveness of the proposed StarPRelu activation function and Enhanced Focal Loss (EFLoss) function in our proposed model, we conduct the ablation study. We set three base models, Base1 (with traditional Relu activation function and CrossEntropy loss function), Base2 (with traditional Relu activation function and our EFLoss function), and Base3 (with our StarPRelu activation function and CrossEntropy loss function). Figure 18 presents the comparison of precision, overall accuracy, and recall between the base models and the HybridSkinFormer model.

In Figure 18, the StarPRelu activation function and Enhanced Focal Loss (EFLoss) function comprehensively improve the model performance. Specifically, the StarPRelu activation function enhances model performance by mitigating the vanishing activation of negative inputs and preventing neuron masking. In contrast, EFLoss ensures the classification accuracy of minority classes in training datasets with imbalanced sample sizes through its loss compensation mechanism.

### 4.4. Comparison

We compare our proposed HybridSkinFormer with several well-known, open-source, and verifiable deep learning models for image classification. To validate the superiority of our approach, we select model variants with comparable parameter counts to ensure a fair comparison. Since all baseline models uniformly support an input format of channels = (r,g,b) and size = (256,256), we resize all input images to 256 × 256 pixels during training and testing the other compared models. Additionally, we adopt the standard Cross-Entropy Loss (CELoss) and use the identical dataset splits (training/testing) as described in this paper. The batch size is fixed at three for all experiments.

The baseline models include:

- Pure ConvNet Architectures: ResNet-20 [9], RegNetY-Small [37], ResNeXt50-32x4d [38], MobileNetV3-Small-1.0 [39], and MobileNetV3-Small-1.0 [39].
- Transformer Architecture-Based Models: DeiT-Tiny/16 [13], PVTv2-B1 [40], MobileViTv3-xxs [41], and CoaT-Tiny [42].

**Figure 19:**
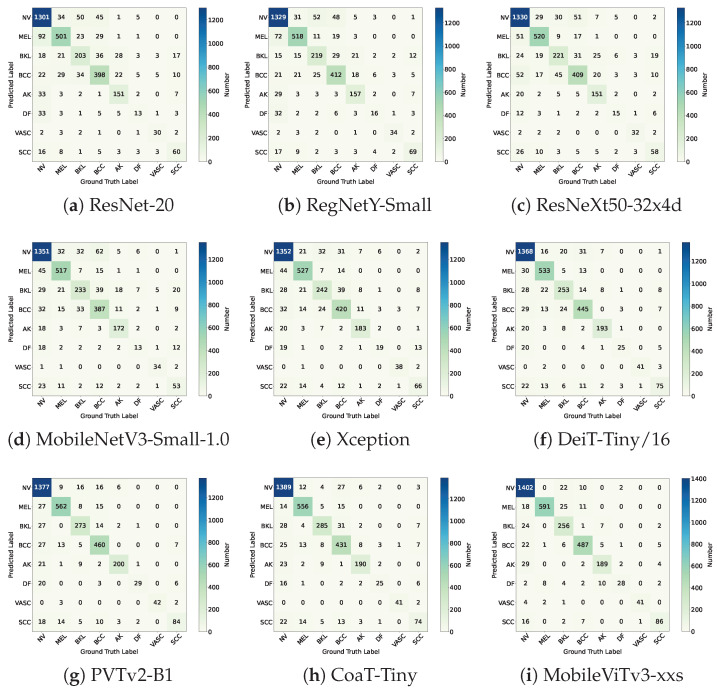
The confusion matrices of the compared deep learning models.

The confusion matrix results for each comparative model are presented in Figure 19.

The overall performance metrics are shown in Table 3. The **bold blue values** represent the best performing indicators and the blue values in regular font represent second best performing indicators. According to the overall performance metrics in Table 3, we can intuitively obtain the following:

- Overall Accuracy: HybridSkinFormer achieved the highest accuracy of 94.2% on the test dataset, outperforming the second-ranked model (MobileViTv3-xxs) by 2%. This demonstrates its superior generalization capability across multi-modality skin lesion images.
- Macro Mean Precision: The model attained a top Macro Mean Precision of 91.1%, exceeding MobileViTv3-xxs (the second-place model) by over 7%. This indicates its enhanced ability to minimize false positive predictions across all lesion classes.
- Macro Mean Recall: HybridSkinFormer achieved a Macro Mean Recall of 91.0%, comparable to MobileViTv3-xxs, highlighting its robustness in detecting positive cases without significant compromise in sensitivity relative to state-of-the-art alternatives.
- Macro Mean F1-Score: The model recorded the highest Macro Mean F1-Score of 0.911, outperforming MobileViTv3-xxs by 0.5 points. This balanced metric reflects its optimal trade-off between precision and recall across imbalanced classes.
- Macro Mean MCC (Matthews Correlation Coefficient): HybridSkinFormer achieved a leading Macro Mean MCC of 0.901, approximately 0.5 points higher than MobileViTv3-xxs. This signifies stronger overall predictive performance, particularly in distinguishing between difficult-to-classify lesion types.

Collectively, HybridSkinFormer demonstrates superior performance across all evaluated metrics, underscoring its accuracy and sensitivity in screening and classifying common skin diseases. These results highlight its clinical utility for early identification of malignant lesions, potentially enabling timely intervention and improving patient outcomes in dermatological diagnostics.

## 5. Conclusions

This work presents HybridSkinFormer, a hybrid deep learning framework that effectively bridges the gap between local and global feature learning for skin lesion classification. By integrating ConvNet and transformer-based attention mechanisms, the model addresses key limitations of single-modality approaches and class imbalance, achieving superior performance across multiple metrics. The introduction of StarPRelu and EFLoss enhances feature representation and training stability, respectively, ensuring robust generalization on diverse datasets. With an overall accuracy of 94.2% and strong macro-averaged scores, HybridSkinFormer demonstrates clinical viability for automated screening, particularly in distinguishing malignant lesions like melanoma (95.5% recall) and basal cell carcinoma (96.3% recall). Future work will focus on enhancing interpretability via attention visualization and validating across broader clinical datasets to further solidify its real-world impact.

## Figures and Tables

**Table 1:** Environment and Parameter Setting.

	CPU: Xeon(R) Platinum 8255C
Hardware	RAM: 40 GB
	GPU: RTX 2080Ti/11 GB with Driver 550.90.07
	OS: Ubuntu 22.04
Software and Library	CUDA: 12.4
	PyTorch: 2.5.1
	batch size: 3,10,20
Training Setting	epoch: 50
	Optimizer: Adam with $\mathrm{learning}\;\mathrm{rate}\;\left(\mathrm{lr}\right)=0.001,\beta_{1}=0.9,\beta_{2}=0.999$
	hidden_dim: 30
	embed_dim: 180
Model Parameters	head_num: 30
	attention_layer_num: 6
	linear bias: True
EFLoss Parameters	β = 3
	γ = 0.3

**Table 2:** Evaluation Metrics of Trained HybridSkinFormer on Test Set.

Class	Overall Accuracy (%)	Precision (%)	Recall (%)	F1-Score	MCC
Melanocytic nevus (NV)	-	97.8	95.6	0.967	0.940
Melanoma (MEL)	-	94.4	95.5	0.950	0.938
Benign keratosis (BKL)	-	86.6	85.8	0.862	0.847
Basal cell carcinoma (BCC)	-	89.0	96.3	0.925	0.912
Actinic keratosis (AK)	-	97.4	90.0	0.936	0.933
Dermatofibroma (DF)	-	78.1	75.8	0.769	0.767
Vascular lesion (VASC)	-	95.1	92.9	0.940	0.939
Squamous cell carcinoma (SCC)	-	91.3	96.0	0.936	0.934
Overall (macro mean)	94.2	91.1	91.0	0.911	0.901

**Table 3:** Performance Comparison to the State-of-the-Art Deep Learning-based Image Classification Models.

Model	Overall Accuracy (%)	Precision (%) (Macro Mean)	Recall(%) (Macro Mean)	F1-Score (Macro Mean)	MCC (Macro Mean)
ResNet-20 [9]	79.6	66.9	69.1	0.676	0.645
RegNetY-Small [37]	82.5	70.7	74.5	0.721	0.695
ResNeXt50-32x4d [38]	81.9	70.4	71.8	0.709	0.681
MobileNetV3-Small-1.0 [39]	82.7	71.1	72.4	0.714	0.688
Xception [43]	85.2	74.9	79.4	0.767	0.745
DeiT-Tiny/16 [13]	87.8	78.0	85.6	0.811	0.795
PVTv2-B1 [40]	90.6	81.4	90.8	0.851	0.841
CoaT-Tiny [42]	89.6	80.1	86.9	0.828	0.815
MobileViTv3-xxs [41]	92.2	83.2	90.5	0.862	0.853
**HybridSkinFormer (our)**	**94.2**	**91.1**	**91.0**	**0.911**	**0.901**

## Data Availability

The datasets generated and/or analyzed during the current study are publicly available: (a) ISIC Challenge 2019 Dataset [https://api.isic-archive.com/collections/65/ (accessed on 8 August 2025)] and [https://api.isic-archive.com/collections/72/ (accessed on 8 August 2025)]. (b) Hospital Italiano de Buenos Aires Skin Lesions Images (2019–2022) Dataset [https://api.isic-archive.com/doi/hospital-italiano-de-buenos-aires-skin-lesions-images-2019-2022/ (accessed on 8 August 2025)]. (c) PAD-UFES-20 Dataset [https://data.mendeley.com/datasets/zr7vgbcyr2/1 (accessed on 8 August 2025)].
